# Risk factors for post-COVID-19 condition: A case-control study in municipalities of Paraíba and Rio de Janeiro, Brazil

**DOI:** 10.1016/j.bjid.2026.105807

**Published:** 2026-03-23

**Authors:** Clarice Monteiro Vianna, Alexandre Medeiros de Figueiredo, Luiz Antonio Bastos Camacho, Celia Menezes Cruz Marques, Janaina Reis Xavier, Vitor Cardoso da Gama, Geovanna de Lima Cunha Pizzini, Renan Marinho Braga, Ewerton Alves Portela dos Santos, Maria de Lourdes de Sousa Maia

**Affiliations:** a Instituto de Tecnologia em Imunobiológicos Bio-Manguinhos (FIOCRUZ), Rio de Janeiro, RJ, Brazil; b Universidade Federal da Paraíba, Departamento de Promoção da Saúde, João Pessoa, PB, Brazil; c Escola Nacional de Saúde Pública (FIOCRUZ), Rio de Janeiro, RJ, Brazil

**Keywords:** Long COVID, Post-COVID-19 condition, Associated factors, Case-control study

## Abstract

**Introduction:**

Post-COVID-19 condition, refers to one or multiple symptoms that continue for several weeks or months following the COVID-19 infection. This study aims to assess the factors associated with post-COVID-19 condition in the Brazilians regions of Paraiba and Rio de Janeiro. Understanding this factor is crucial to public policies and improving care for high-risk individuals.

**Methods:**

This is a multicenter, retrospective case-control study. Cases were classified as individuals experiencing persistent symptoms for over 12-weeks following a confirmed COVID-19 diagnosis, whereas controls were those who recovered. Data on sociodemographic characteristics, medical history, lifestyle factors, and vaccination status were gathered, and logistic regression was used to analyze the relationship between these variables and the onset of post-COVID-19 condition.

**Results:**

Of the 1.350 participants, 734 were cases and 616 were controls, with a 54% frequency of post-COVID-19 condition. The study found significant associations between the syndrome and female sex (OR = 2.09; 95% CI 1.6‒2.74), middle age (OR = 2.03; 95% CI 1.31‒3.15 for 40- to 60-years) and over 60 (OR=1.84; 95% CI 1.06‒3.2), white people (OR = 1.41; 95% CI 1.07‒1.86), hypercholesterolemia (OR = 1.66; 95% CI 1.18‒2.32), chronic lung diseases (OR = 5.60; 95% CI 1.18‒26.65), mental illness such as anxiety and panic disorder (OR = 2.52; 95% CI 1.64‒3.87), family history of long COVID syndrome (OR = 2.07; 95% CI 1.45‒2.97), hospitalization (OR = 5.89; 95% CI 3.92‒8.86) and good pre-COVID health perception (OR = 0.69; 95% CI 0.53‒0.9).

**Conclusions:**

The findings reinforce the importance of identifying groups more frequently affected by post-COVID-19 condition and supporting follow-up strategies aimed at mitigating its impact on individuals and health services.

## Introduction

In May 2023, with evidence of a significant decline in the weekly number of COVID-19 related deaths, hospitalizations, and Intensive Care Units (ICU) admissions, the World Health Organization (WHO) determined the end of the Public Health Emergency of International Concern for COVID-19.^1^ Despite the end of the pandemic, individuals who have had COVID-19 may experience sustained post-infection sequelae. This condition, known by various names such as long COVID, post-acute COVID-19, post-acute sequelae of SARS-CoV-2 infection, post-COVID conditions, long-term effects of COVID, chronic COVID, is classified in the International Classification of Diseases, 11th Revision (ICD-11) as post-COVID-19 condition.^2^

Major international public health agencies have standardized the definitions and terminology of this condition. The National Institute for Health and Care Excellence (NICE), in their COVID-19 rapid guideline on managing the long-term effects of COVID-19, categorizes post-COVID-19 syndrome as signs and symptoms developing during or after an infection consistent with COVID-19, persisting for more than 12-weeks and not explained by an alternative diagnosis.^3^ The World Health Organization, in October 2021, established a clinical case definition for post-COVID-19 condition: “post-COVID-19 condition occurs in individuals with a history of probable or confirmed SARS-CoV-2 infection, usually 3-months from the onset of COVID-19, with symptoms that last for at least 2-months and cannot be explained by an alternative diagnosis. Common symptoms include fatigue, shortness of breath, cognitive dysfunction, but also others, and generally have an impact on everyday functioning. Symptoms may be new onset following initial recovery from an acute COVID-19 episode or persist from the initial illness. Symptoms may also fluctuate or relapse over time”.^4^

Post-COVID-19 syndrome can cause symptoms across multiple systems, such as respiratory (shortness of breath, cough), cardiovascular (chest tightness, palpitations), general symptoms (fatigue, fever, pain), neurological symptoms (cognitive impairment, headache, sleep disturbances, musculoskeletal symptoms (joint and muscle pain), as well as psychological/psychiatric symptoms (depression, anxiety).^3^ Over 200 symptoms have been reported, with an estimated 65 million individuals worldwide experiencing long COVID, with cases increasing daily.^5^

The prevalence of long COVID varies widely in literature due to different definitions and symptom duration considerations. A 2022 study found 21.8 % of adults had symptoms lasting more than 3-months, with younger age, women, low income, overweight or obesity, smokers or ex-smokers, individuals with depression, and unvaccinated adults being more likely to experience long COVID.^6^ O’Mahoney and colleagues found that in a 2023 systematic review the pooled prevalence of COVID-19 survivors experiencing at least one symptom at follow-up was 52.6 % in hospitalized patients and 34.5 % in the non-hospitalized group.^7^ A 2025 systematic review and meta-analysis estimated that more than half of the COVID-19 survivors (56.5 %) reported experiencing one or more symptoms from 12- to 26-weeks. Fatigue and dyspnea were the most common symptoms reported, followed by headaches, myalgia, and chest pain. Limitations in returning to work were the most reported outcomes related to functional impairment in studies.^8^

The present study aims to estimate the frequency and assess the factors associated with post-COVID-19 syndrome in the population residing in municipalities in Paraiba and in the municipality of Rio de Janeiro. The results may help in the clinical investigation and management of nonspecific signs and symptoms that comprise the syndrome and thus provide more effective support to people with the condition. Additionally, identifying groups that may be at higher risk can contribute to the development of strategies and guide specific care for those who might suffer long-term effects.

## Material and methods

### Study design

This is a multicenter, retrospective, case-control study strategically planned and structured by the Department of Medical Affairs, Clinical Studies, and Post-Marketing Surveillance (DEAME) (Bio-Manguinhos – Fiocruz) to assess the factors associated with post-COVID-19 condition in municipalities of Paraíba (Alhandra, Caaporã, Conde, João Pessoa) and in the municipality of Rio de Janeiro. Case finding among individuals with confirmed COVID-19 infection according to the Ministry of Health criteria^9^ also allowed estimation of the frequency of post-COVID-19 condition. The main criteria used in the definition of cases for the primary outcome was the temporal of 3-months (12-weeks) and the clinical symptomatology of persistent symptoms (one or more symptoms) in individual with a history of COVID-19, as outlined in the Delphi consensus by the WHO,^4^ and by the National Institute for Health and Care Excellence (NICE), Scottish Intercollegiate Guidelines Network (SIGN), and Royal College of General Practitioners (RCGP),^3^ as post-COVID-19 Condition/Post-COVID-19 Syndrome, respectively. The symptoms included in the questionnaire of the study were those indicated in the NICE guidelines^3^ and the Delphi consensus by WHO (> 18-years),^4^ and for children and adolescents (< 18-years) those described in the Meta analysis “Long-COVID in children and adolescents: a systematic review and meta-analyses”.^10^

Individuals meeting the Ministry of Health's criteria for a COVID-19 diagnosis^9^ were assessed retrospectively. These COVID-19 infected individuals were identified as cases, when there were one or more signs/symptoms with a duration of more than twelve weeks consistent with post-COVID-19 Condition, and as controls those who did not develop the condition.

The cases and controls were compared in order to estimate the association with sociodemographic characteristics (age, sex, race, schooling), medical history (pre-existing conditions, obesity), lifestyle (smoking, alcohol consumption, physical activity/sedentary behavior), factors related to the course of acute COVID-19 infection (hospitalization, impact on work and leisure activities), family history of post-COVID-19 condition, blood type, vaccination status at the time of the COVID-19 diagnosis (including the number of doses), and the predominant circulating variant at the time of acute infection.

Inclusion criteria required participants to be between 0- and 80-years old and to have either a laboratory-confirmed diagnosis of COVID-19 or to have been reported as a confirmed case of COVID-19 in the SUS Health Surveillance Database (e-SUS Notifica) or in the Epidemiological Surveillance System of Influenza (SIVEP-Gripe), according to the criteria set by the Brazilian Ministry of Health. Any behavioral, cognitive, or psychiatric disorder that impaired the ability to understand and comply with the study was an exclusion criterion. Participants from the SIVEP-Gripe database who were listed as deceased were excluded.

The e-SUS Notifica and SIVEP-Gripe databases were requested from the Municipal Health Secretariats of Alhandra, Caaporã, Conde, João Pessoa, and Rio de Janeiro, as well as from the State Health Secretariat of Paraíba. Confirmed COVID-19 cases in individuals up to 80-years-old, occurring between March 2020 and January 2024 in Paraiba and January 2023 to November 2023 in Rio de Janeiro, were selected according to the study's inclusion criteria. After this selection, a list was created with eligible individuals for the study, including those with records allowing telephone contact. Recruitment took place between 09/01/2023 and 18/07/2024. The first individuals invited to participate in the study were participants and cohabitants from another Bio-Manguinhos ‒ Fiocruz study in the municipalities of Caaporã, Conde, and Alhandra in Paraíba, titled: “Immunity Against Yellow Fever After a Single Vaccine Dose in Children and Adults: A Cohort Study in a Non-Endemic Area”. Subsequently, recruitment was expanded to include any reported case in these same municipalities and later extended to João Pessoa and Rio de Janeiro.

The initial contact was made by phone or through apps (SMS, WhatsApp). Then a personal visit was scheduled to present details and to get a signed Informed Consent Form or an Assent Form. Participants were then interviewed using a structured clinical-epidemiological questionnaire with sociodemographic data, medical history, vaccination history, and current clinical information recorded at the time of study enrolment. Vaccination status was recorded only when documented on vaccination cards. To classify participants as CASES (persistent symptoms for more than 12-weeks) or CONTROLS, the questionnaire included questions about the evaluation of signs and symptoms after the acute phase to assess the study's primary outcome. The first episode of COVID-19 for each participant, recorded in the national notification system databases, the first positive laboratory test, or the date of symptom onset, in that order of preference, was considered.

### Statistical analysis

Sample size calculations were performed, assuming a 95 % confidence level and 80 % statistical power, aiming to detect odds ratios for the association between specific factors associated with the occurrence of post-COVID-19 condition. The estimates were based on a case-control design comparing exposed and unexposed individuals. To ensure sufficient statistical power across all comparisons, the largest sample size estimated for the different exposures was 800 cases and 800 controls. Simulations were performed under a range of scenarios, varying both the proportion of exposure among controls (from 5 % to 90 %) and corresponding odds ratios. These scenarios ensured that adequate power would be maintained even in configurations with slightly smaller sample sizes. For each combination of exposure prevalence and odds ratio, a minimum required sample size for cases and controls was determined to guide the final sample estimation. Frequency matching of controls was implemented for the age groups 0- to 19-years, 20- to 39-years, 40- to 60-years, and over 60-years.

The entire sample was characterized considering the case and control subgroups and presented in frequency distribution tables. Associations between the outcome (post-COVID-19 condition) and explanatory variables were assessed by estimating odds ratios comparing cases and controls. Multivariable logistic regression analyses were performed using a stepwise variable selection strategy that considered both statistical criteria and the scientific relevance of covariates, under the supervision of a statistician. A significant level of 95 % was adopted, and the statistical analyses were conducted using IBM SPSS Statistics 20.0 software (IBM Corp. Released, 2011).

### Ethics

The project was approved by the Research Ethics Committees of the Lauro Wanderley University Hospital (opinion n° 5.813.351), the State Health Secretariat of Paraíba (opinion n° 6.095.451) and the Municipal Health Secretariat of Rio de Janeiro SMS/RJ (opinion n° 6.006.719).

## Results

**Fig. 1:**
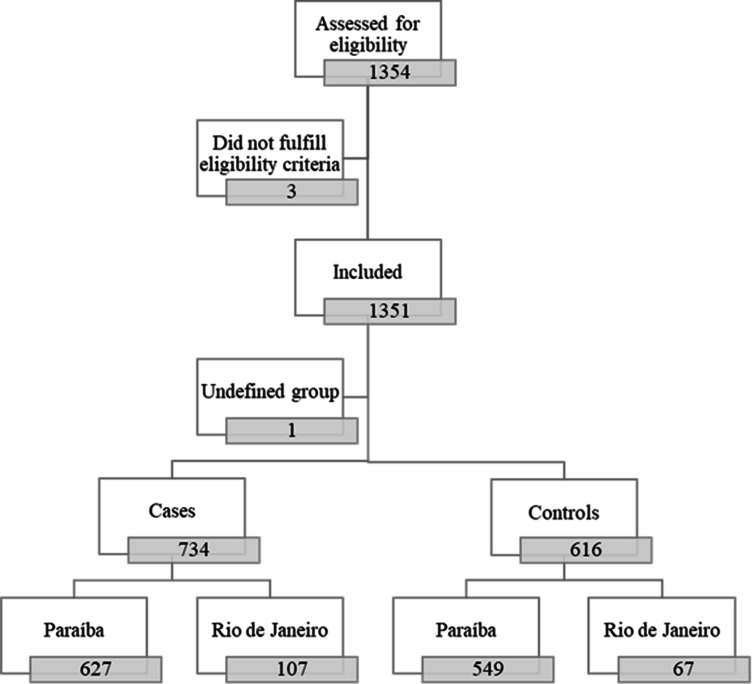
Consort diagram.

The results of 1.350 participants were analyzed, divided into 734 cases and 616 controls, resulting in a post-COVID-19 syndrome frequency of 54 %. Most of the sample were from the state of Paraíba (Fig. 1).

Females, middle-aged, black/brown, and less educated individuals comprised higher proportions of participants. Nevertheless, females, middle-aged, white and more educated appeared more likely to have post-COVID-19 condition (Table 1).

**Table 1:** Participant’s characteristics and unadjusted estimates of association (simple logistic regression).

Participants Characteristics	Cases n (%)	Controls n (%)	OR (95 % IC)	p
Sex				
Male	238 (32.4)	283 (45.9)	1	
Female	496 (67.6)	333 (54.1)	1.77 (1.42‒2.21)	0.000
Age, years				
0‒19	46 (6.3)	104 (16.9)	1	
20‒39	219 (29.8)	221 (35.9)	2.24 (1.51‒3.32)	0.000
40‒60	363 (49.5)	223 (36.2)	3.68 (2.5‒5.41)	0.000
> 60	106 (14.4)	68 (11.0)	3.52 (2.22‒5.59)	0.000
Race				
Black and Brown	468 (64.5)	448 (73.0)	1	
White	258 (35.5)	165 (27.0)	1.50 (1.18‒1.89)	0.001
Educational level				
No education/ Elementary/ High school^a^	434 (59.1)	448 (72.7)	1	
Higher education^b^	300 (40.9)	168 (27.3)	1.84 (1.46‒2.32)	0.000
Medical History^c^				
Diabetes	89 (12.1)	36 (5.8)	2.22 (1.49‒3.33)	0.000
Hypertension	210 (28.6)	100 (16.2)	2.07 (1.58‒2.7)	0.000
Cardiopathy	42 (5.7)	24 (3.9)	1.50 (0.9‒2.5)	0.123
Hypercholesterolemia	198 (27.0)	83 (13.5)	2.39 (1.8‒3.17)	0.000
Chronic Kidney disease	30 (4.1)	8 (1.3)	3.24 (1.47‒7.12)	0.003
Cerebrovascular accident	10 (1.4)	11 (1.8)	0.76 (0.32‒1.8)	0.535
Asthma or asthmatic bronchitis	54 (7.4)	29 (4.7)	1.61 (1.01‒2.56)	0.045
Another chronic lung disease^d^	22 (3.0)	2 (0.3)	9.49 (2.22‒40.5)	0.002
Rheumatism and/or autoimmunities^e^	52 (7.1)	22 (3.6)	2.06 (1.24‒3.43)	0.006
Depression	83 (11.3)	22 (3.6)	3.43 (2.11‒5.55)	0.000
Other Mental illness^f^	121 (16.5)	41 (6.7)	2.76 (1.9‒4)	0.000
Skin cancer	10 (1.4)	6 (1.0)	1.40 (0.51‒3.89)	0.513
Any other type of cancer	16 (2.2)	8 (1.3)	1.69 (0.72‒3.98)	0.227
Coagulation disorders^g^	20 (2.7)	4 (0.6)	4.29 (1.46‒12.61)	0.008
Difficulty in walking	30 (4.1)	15 (2.4)	1.70 (0.91‒3.2)	0.097
Other chronic disease (>6-months)	164 (22.3)	85 (13.8)	1.80 (1.35‒2.4)	0.000
Obesity (adults BMI ≥30)	262 (38.8)	160 (31.1)	1.41 (1.1‒1.79)	0.006
Blood type				
Type O	248 (47)	203 (52)	1	
Type A or B or AB	282 (53)	190 (48)	1.21 (0.94‒1.58)	0.144

a No formal education, incomplete/completed elementary or incomplete/completed high school.

b Higher education: Incomplete/completed higher education or postgraduate studies.

c Reference category for the OR: not having the specified disease, but possibly having other diseases.

d Such as emphysema, chronic bronchitis, or COPD (Chronic Obstructive Pulmonary Disease).

e Lupus, rheumatoid arthritis, ankylosing spondylitis.

f Such as anxiety disorder, panic disorder, schizophrenia, bipolar disorder, psychosis, or OCD (obsessive-compulsive disorder).

g Thrombosis, thrombophilia, hemophilia, or other diseases that impair blood clotting.

Regarding the clinical conditions prior to COVID-19, participants were asked about the diagnosis by a doctor, or in the case of mental illnesses, by a doctor or psychologist. The most prevalent clinical condition in cases and controls before COVID-19 was hypertension (28.6 % in cases and 16.2 % in controls), followed by hypercholesterolemia (27 % in cases and 13.5 % in controls), and mental illness such as anxiety disorder and panic disorder (16.5 % in cases and 6.7 % in controls). Almost 23 % of cases had chronic long-term illnesses whereas almost 14 % of controls lived with chronic health conditions. Overweight and obesity were frequent and appeared somewhat more likely to develop post-COVID-19 condition. Chronic lung diseases (other than asthma), coagulation disorders, depression, and chronic kidney disease were more strongly associated with post-COVID-19 condition (Table 1).

Almost 50 % of cases and controls engaged in some form of physical activity before contracting the COVID-19 infection. A small proportion were smokers, and one-quarter consumed alcoholic beverages. None of those lifestyle elements appeared associated with post-COVID-19 condition. More than half of cases and controls considered their health to be good before the COVID-19 infection and were less likely to develop post-COVID-19 condition. A substantial proportion of participants reported a family history of post-COVID-19 symptoms (30.6 %) or hospitalization (20.6 %), which appeared to increase the odds of developing that syndrome (Table 2).

**Table 2:** Exposure profile and unadjusted association with post-COVID-19 condition (simple logistic regression).

Exposure profile	Cases n ( %)	Controls n ( %)	OR (95 % IC)	p
Habits/ Medications				
Engagement in any physical activity^a^	353 (48.1)	287 (46.6)	1.06 (0.86‒1.32)	0.585
Walking or using a bicycle to work	118 (16.1)	103 (16.7)	0.96 (0.72‒1.28)	0.758
Smoking (4 weeks pre-COVID-19)	30 (4.1)	18 (2.9)	1.42 (0.78‒2.58)	0.246
Alcohol use before COVID-19	198 (27.0)	151 (24.5)	1.14 (0.89‒1.45)	0.303
Taking any medication	318 (43.3)	167 (27.1)	2.07 (1.64‒2.6)	0.000
Pre-COVID-19 health perception				
Excellent	133 (18.1)	132 (21.4)	1	
Very good	150 (20.4)	106 (17.2)	1.40 (0.99‒1.99)	0.054
Good	418 (56.9)	369 (59.9)	1.12 (0.85‒1.49)	0.410
Bad/Very bad	33 (4.6)	9 (1.5)	3.64 (1.68‒7.9)	0.001
Vaccine regimen types^b^				
No doses	581 (79.3)	469 (76.1)	1	
One single dose	31 (4.2)	21 (3.4)	1.19 (0.68‒2.1)	0.545
Complete primary regimen	62 (8.5)	62 (10.1)	0.81 (0.56‒1.17)	0.260
Complete primary + ≥ 1 booster	59 (8.0)	64 (10.4)	0.74 (0.51‒1.08)	0.122
Family History^c^				
Hospitalization due to COVID-19	101 (13.8)	42 (6.8)	2.19 (1.5‒3.19)	0.000
Death due to COVID-19	47 (6.4)	27 (4.4)	1.49 (0.92‒2.43)	0.105
Post-COVID-19 symptoms > 3 months	150 (20.4)	63 (10.2)	2.27 (1.66‒3.12)	0.000
Variant^d^^,^^e^				
XBB (Omicron) and B.1.617.2 (Delta)	145 (21)	180 (30.1)	1	
B1.1.28 (Beta) and B1.1.33	224 (32.4)	185 (30.9)	1.50 (1.12‒2.01)	0.006
B.1.1.7 (Alfa) and P2 (Zeta)	86 (12.4)	63 (10.5)	1.69 (1.15‒2.51)	0.008
P1 (Gama)	236 (34.2)	171 (28.5)	1.71 (1.28‒2.3)	0.000
Hospitalization data				
Required hospitalization	223 (30.4)	39 (6.3)	6.49 (4.53‒9.31)	0.000
Required Intensive Care Unit^f^	78 (35.0)	8 (20.5)	2.08 (0.91‒4.75)	0.081
Required intubation^f^	22 (9.9)	1 (2.6)	4.16 (0.54‒31.79)	0.170
Required Hemodialysis^f^	7 (3.1)	1 (2.6)	1.23 (0.15‒10.3)	0.848
Needed oxygen at home^f^	21 (9.4)	1 (2.6)	3.95 (0.52‒30.25)	0.186

a To improve health, physical condition, or for esthetic or leisure purposes.

b One participant was left out of this categorization due to inconsistent information.

c Father, mother, sibling, or biological children.

d 43 case participants did not know/remember the onset date of symptoms, as well as 17 controls.

e Circulating variant on the onset date of symptoms.

f Data only from hospitalized participants.

Nearly four times as many cases as controls were hospitalized due to COVID-19 and the frequency of post-COVID-19 conditions was 85 % among these participants who needed hospital care. Participants who required hospitalization had higher odds of presenting symptoms lasting longer than twelve weeks (Table 2). Besides, more than three-fourths of both cases and controls had not been vaccinated before the infection and vaccination showed no association to post-COVID-19 condition.

The most frequent circulating variants at the onset of symptoms among cases were Beta and Gama that were reported in Brazil in 2020 and the first half of 2021 respectively.^11^ In the univariable logistic regression analysis, it was observed that when the onset of COVID-19 symptoms coincided with the circulation of pre-Delta and pre-Omicron variants, the chance of developing post-COVID-19 syndrome increased by 1.5 times for the Beta variant, 1.69 times for Alpha, and 1.71 times for Gamma (Table 2).

**Fig. 2:**
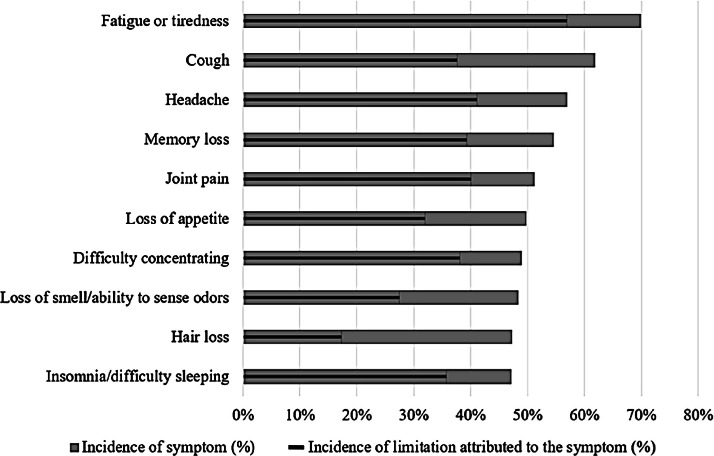
Distribution of leading symptoms and their impact on daily functional capacity.

As shown in Fig. 2, the highest frequencies were observed in the following symptoms in case participants: fatigue (70 %), cough (62 %), headache (57 %), memory loss (54 %), joint pain (51 %), loss of appetite (50 %), and difficulty concentrating (49 %). It was also noted that fatigue, joint pain, and difficulty concentrating caused significant limitations in daily activities for those who experienced these symptoms. Overall, general, respiratory and neurological symptoms were the most frequent.

The multivariate logistic regression (Table 3) demonstrated that the variables most strongly associated with post-COVID-19 condition were hospitalization and chronic lung disease. Mental illness, family history of post-COVID-19 condition, age above 40-years and female sex were also significant predictors. Race, self-perception of health before COVID-19 and hypercholesterolemia showed association of low magnitude. Chronic kidney disease showed a marginal association. Vaccination status before COVID-19 infection was not associated to post-COVID-19 condition.

**Table 3:** Adjusted measures from the final multivariable logistic regression model.

Variable	OR (95 % IC)	p-value
Constant	0.21	‒
Sex		
Male	1	‒
Female	2.09 (1.6‒2.74)	0.000
Age group		
0 to 19 years	1	‒
20 to 39 years	1.43 (0.92 ‒2.22)	0.110
40 to 60 years	2.03 (1.31‒3.15)	0.002
Over 60 years	1.84 (1.06‒3.2)	0.031
Race/Ethnicity		
Black and Brown	1	‒
White	1.41 (1.07 ‒1.86)	0.014
Perception of health before COVID-19		
Excellent/Very good	1	‒
Good	0.69 (0.53 ‒0.9)	0.006
Bad or very bad	1.34 (0.56‒3.16)	0.510
Clinical condition		
Hypercholesterolemia	1.66 (1.18‒2.32)	0.003
Chronic kidney disease	2.31 (0.97‒5.48)	0.058
Another chronic lung disease^a^	5.60 (1.18‒26.65)	0.030
Other Mental illness^b^	2.52 (1.64‒3.87)	0.000
Hospitalization data		
Required hospitalization	5.89 (3.92‒8.86)	0.000
Family history		
Family history^c^ of problems after COVID-19 for more than 3-months	2.07 (1.45‒2.97)	0.000
Vaccination status before COVID-19 infection		
Not vaccinated	0.96 (0.7‒1.3)	0.779

a Such as emphysema, chronic bronchitis, or COPD (Chronic Obstructive Pulmonary Disease.

b Such as anxiety disorder, panic disorder, schizophrenia, bipolar disorder, psychosis, or OCD (Obsessive-Compulsive Disorder).

c Father, mother, sibling, or biological children.

## Discussion

In a sample of individuals who had been diagnosed with COVID-19 in several municipalities in the state of Paraíba and in the city of Rio de Janeiro, we found that 54 % developed the post-COVID-19 condition. History of chronic diseases, mental illness, hospitalization for COVID-19, female sex, age of 40-years or more and family history of post-COVID-19 condition were the factors most strongly associated with post-COVID-19 condition after multivariable adjustment.

The frequency of post-COVID-19 syndrome observed in this study is consistent with findings from meta-analysis and systematic reviews.^12^^,^^13^ Reported prevalence varies widely in the literature, depending on case definition, the timing of data collection, follow-up duration, severity of acute infection, methods of symptom ascertainment and sample characteristics.^14^^,^^15^

This relatively high frequency may be explained by several factors. First, the case definition adopted followed WHO and NICE criteria, which prioritize sensitivity and consider the presence of at least one persistent symptom. In addition, markers of greater severity of acute COVID-19 such as the need for hospitalization, were associated with post-COVID-19 condition in this cohort, suggesting that the disease severity may have contributed to the overall burden of persisting symptoms. Finally, most infections occurred during the circulation of more virulent variants, which may have further influenced the observed frequency.

Post-COVID-19 condition is a multisystem disorder involving pathophysiological mechanisms, including microthrombi, direct virus-induced damage, and immune-mediated injurie.^14^ In this study fatigue was the most frequent reported symptom, consistent with previous literature.^13^^,^^14^^,^^16^ Persistent fatigue is a well-recognized sequela of infectious diseases and likely reflects a complex interaction of immunological and psychosocial factors.^17^ It impacts daily functioning, word capacity, and quality of life.

Consistent with findings from systematic reviews and meta-analyses^13^^,^^18^ respiratory, neurological, and psychological symptoms were among the most frequently reported manifestations. In this study, fatigue, respiratory symptoms, and cognitive complaints predominated.

Female sex has been consistently associated with post-COVID-19 condition in the literature.^18^, ^19^, ^20^, ^21^, ^22^, ^23^, ^24^ This association may reflect sex-related differences in immune and hormonal responses ‒ such as higher IgG antibody production during the acute response, which may result in a more favorable outcome during the disease onset, but could be associated with the persistence of disease manifestations – as well as social and behavioral factors, including greater symptom awareness and disproportionate psychosocial burden during the pandemic.^24^, ^25^, ^26^ In line with what has been observed in the literature, in this study, female sex had a higher likelihood of developing post-COVID-19 condition.

Statistically significant association of post-COVID-19 condition was observed among middle aged (40‒60-years) and over 60. Although evidence regarding age remains heterogeneous, several studies report higher frequencies of persistent symptoms in middle-aged and older adults,^27^, ^28^, ^29^, ^30^, ^31^, ^32^ possibly reflecting cumulative comorbidities. Several studies pointed out that post-COVID-19 condition impacted quality of life, loss of work capacity, and functionality of individuals.^33^, ^34^, ^35^ In this perspective, the involvement of groups within the economically active population, such as middle-aged individuals, points to the social and economic consequences of the syndrome that need further analysis.

It is described in literature that the COVID-19 pandemic has disproportionately affected minority groups, including African American, Native American, and Latin communities, due to factors like chronic conditions, limited healthcare, and poor living conditions.^36^^,^^37^ In this study we found a higher risk in white race. These results resemble those found in large retrospectives studies,^27^^,^^30^ but it should be interpreted cautiously, as residual confounding, differential access to diagnosis, and reporting bias that cannot be excluded.

A systematic review and meta-analysis, Tsampasian and colleagues reported that comorbidities were associated with a higher risk of developing post-COVID-19 condition.^28^ Anxiety and other mental illnesses have also been consistently associated with the condition^22^^,^^27^^,^^38^^,^^39^ and frequently persist as long-term symptoms.^22^^,^^23^ The pandemic itself could affect mental health due to its significant impact on the societies and economies of countries, increasing risk factors such as unemployment, grief, and financial insecurity.^40^

The Pan American Health Organization, through its High-Level Commission on Mental Health and COVID-19, has provided guidance to countries to improve mental health care, such as integrating mental health into all policies, improving and expanding mental health services throughout the life cycle, and enhancing data and research in mental health.^41^ Therefore, this work provides further evidence on the need for all government sectors to ensure that mental health is addressed at all levels of care and prevention.

Poorer self-rated health prior to COVID-19, although only the “good” category showed a statistically significant association, suggests that self-perceived health status may play a role in vulnerability to the outcome. Consistent with previous studies, a prior history of chronic lung disease and hypercholesterolemia were associated with the condition^22^^,^^27^^,^^28^^,^^42^^,^^43^, however the former finding should be interpreted with caution due to the small number of exposed participants.

To our knowledge, the association between family history and post-COVID-19 have not been previously reported. In this study, having first-degree relatives with persistent post-COVID-19 symptoms was associated with a higher likelihood of developing the condition. This finding is biologically plausible and may reflect genetic susceptibility,^44^ which could bring this familial tendency to the disease, such as the association of certain HLA-I alleles that have lower affinity for SARS-CoV-2 antigens and could contribute to the development of post-COVID-19 condition by inducing less effective immune responses.^45^

Hospitalization during acute COVID-19 was strongly associated with post-COVID-19 condition. These data are consistent with previous studies,^22^^,^^28^^,^^46^ which highlighted that patients with prior critical illnesses are a high-risk population that should be monitored and have prevention and rehabilitation strategies implemented.

Vaccination status prior to SARS‑CoV‑2 infection was not significantly associated with the outcome in this study in contrast to findings from multiple systematic reviews indicating that full vaccination reduces the risk of long COVID compared to unvaccinated individuals.^47^ This result may be explained by the high proportion of unvaccinated participants at the time of infection and by infections occurring predominantly during earlier pandemic waves, when clinical protocols were less developed, and many participants being infected before widespread of vaccine availability ‒ potentially confounding the observed null association and limiting the ability to detect a protective effect.

Some limitations should be acknowledged. Because data on symptoms, lifestyle factors, and pre-COVID health status were self-reported, some degree of recall bias and misclassification cannot be excluded, particularly given the interval between infection and interview for some participants. Although the possibility that some controls may have developed symptoms after the assessment period cannot be excluded, this issue was considered in the interpretation of the findings and is unlikely to have materially influenced the results. In addition, changes in the magnitude of associations after multivariable adjustment highlight the importance of cautious interpretation of univariate findings. Finally, the use of a convenience sampling strategy may limit generalizability.

## Conclusions

Post-COVID-19 condition was a sequela left by the pandemic, and it will be the subject of studies for a long time to better understand its etiology, the correlation between selected symptoms and the condition, factors associated, markers, treatment, and impacts on global public health.

This case-control study identified factors statistically associated with post-COVID-19 condition. Female sex, middle age, and white race were associated with a higher likelihood of the syndrome. Pre-existing conditions such as hypercholesterolemia, chronic lung diseases, and mental health disorders also were associated with an increased likelihood of the condition. Furthermore, a family history of the condition and previous hospitalization due to COVID-19 emerged as significant contributing factors.

These findings are in line with the most recent literature and suggest that these groups may benefit from closer follow-up after recovery, considering the impact on quality of life and long-term health. Moreover, the history of COVID-19 should be considered in the clinical investigation and management of the nonspecific signs and symptoms shared by multiple etiologies.

Furthermore, the factors associated identified in this study may contribute to developing strategies to reduce the impact on patient’s quality of life and on the health system.

## Conflicts of interest

The authors declare no conflicts of interest.

## Data Availability

The data that support the findings of this study are available from the corresponding author upon reasonable request.
